# Surgical Treatment for Congenital Heart Defects in Down Syndrome Patients

**DOI:** 10.21470/1678-9741-2018-0358

**Published:** 2019

**Authors:** Fernando Cesar Gimenes Barbosa Santos, Ulisses Alexandre Croti, Carlos Henrique De Marchi, Alexandre Noboru Murakami, Juliana Dane Pereira Brachine, Bruna Cury Borim, Renata Geron Finoti, Moacir Fernandes de Godoy

**Affiliations:** 1 Serviço de Cardiologia e Cirurgia Cardiovascular Pediátrica de São José do Rio Preto - Hospital da Criança e Maternidade de São José do Rio Preto (FUNFARME) - Faculdade de Medicina de São José do Rio Preto (FAMERP), São José do Rio Preto, SP, Brazil.; 2 Serviço de Cirurgia Cardíaca do Norte do Paraná, Universidade Estadual de Londrina (UEL), Londrina, PR, Brazil.

**Keywords:** Down Syndrome, Heart Defects, Congenital, Database

## Abstract

**Objective:**

To analyze data related to surgical treatment in patients with congenital heart defects (CHD) and Down syndrome (DS) based on information from International Quality Improvement Collaborative Database for Congenital Heart Disease (IQIC).

**Methods:**

Between July 1, 2010 and December 31, 2017, 139 patients with CHD and DS underwent surgery at Hospital de Base and Hospital da Criança e Maternidade de São José do Rio Preto (FUNFARME)/Faculdade de Medicina de São José do Rio Preto - SP (FAMERP). A quantitative, observational and cross-sectional study was performed in which the pre, intra and postoperative data were analyzed in an IQIC database. The data included gender, age, prematurity, weight, preoperative procedures, diagnosis, associated cardiac and non-cardiac anomalies, Risk Adjustment for Congenital Heart Surgery (RACHS-1), type of surgery, cardiopulmonary bypass (CPB), perfusion time, aortic clamping time and CPB temperature, bacterial sepsis, surgical site infection and other infections, length of stay in intensive care unit (ICU), length of hospital stay and in-hospital mortality.

**Results:**

The most prevalent procedures were complete atrioventricular septal defect repair (58 - 39.45%), followed by closure of ventricular septal defect (36 - 24.49%). The RACHS-1 categories 1, 2, 3 and 4 were distributed as 22 (15%); 49 (33.3%); 72 (49%) and 4 (2.7%), respectively. There were no procedures classified as categories 5 or 6. Bacterial sepsis occurred in 10.2% of cases, surgical site infection in 6.1%, other infections in 14.3%. The median length of ICU stay was 5 days and the median length of hospital stay was 11 days. In-hospital mortality was 6.8%.

**Conclusion:**

Surgical treatment in patients with CHD and DS usually does not require highly complex surgical procedures, but are affected by infectious complications, resulting in a longer ICU and hospital length of stay with considerable mortality.

**Table:** 

Abbreviations, acronyms & symbols	
AVSD	= Atrioventricular septal defect
CHD	= Congenital heart defects
CPB	= Cardiopulmonary bypass
DS	= Down syndrome
ES	= Eisenmenger syndrome
ICU	= Intensive care unit
IQIC	= International Quality Improvement Collaborative
	Database for Congenital Heart Disease
PAH	= Pulmonary arterial hypertension
RACHS-1	= Risk Adjustment for Congenital Heart Surgery

## INTRODUCTION

Congenital heart defects (CHD) are present in patients with Down syndrome (DS) or trisomy 21 in approximately 40-50% of cases^[1,2]^. Among the most common cardiac defects are atrioventricular septal defect (AVSD), representing approximately 45% of cases, and ventricular septal defect (VSD), representing 20-30%^[1,3]^.

The surgical treatment of CHD in these patients usually involves greater risk of postoperative complications and mortality^[2]^. Adequate pre, intra and postoperative control can be obtained with a reliable database. In our environment, data is stored in a database entitled International Quality Improvement Collaborative for Congenital Heart Disease (IQIC)^[4]^.

This international database, designed by Dr. Kathy J. Jenkins of Boston Children's Hospital - Harvard Medical School, aims to reduce mortality and complications within 30 days of the procedure associated with pediatric cardiovascular surgery in developing countries^[5]^.

Thus, we planned to analyze data related to surgical treatment in patients with CHD and DS based on the information available in the IQIC database.

## METHODS

From July 1 2010 to December 31 2017, 1,284 patients were operated at Hospital de Base and at Hospital da Criança e Maternidade de São José do Rio Preto (FUNFARME)/Faculdade de Medicina de São José do Rio Preto - SP (FAMERP) and included into the IQIC database, of which 139 (10.83%) were patients with CHD and DS.

A quantitative, observational and cross-sectional study was carried out in which pre, intra and postoperative data of the 139 patients with CHD and DS included in the IQIC database who underwent 147 surgical procedures were analyzed.

The information was collected through patients' electronic medical record and sent via REDCap(r) platform to Boston Children's Hospital group at Harvard Medical School, which periodically audits data through a face-to-face or Web-based visit to identify possible failures. The data are analyzed and reported every six months to the participating centers by the IQIC group, being compared with other participating sites and within the center itself.

Preoperative data included gender, age at time of operation, history of prematurity, weight, preoperative procedures, diagnosis, and presence of associated cardiac and non-cardiac anomalies.

Intraoperative data included procedures Risk Adjustment for Congenital Heart Surgery (RACHS-1), which ranks categories from 1 (lowest risk) to 6 (highest risk) and is a useful tool for predicting the risk of mortality^[6]^.

Surgical descriptions of each patient were analyzed observing the type of surgery, use of cardiopulmonary bypass (CPB), perfusion time, aortic clamping time and temperature during CPB.

Post-operative data analysis included complications such as bacterial sepsis, surgical site infection and other infections such as necrotizing enterocolitis, tracheitis and pneumonia associated or not with mechanical ventilation, length of hospital stay in intensive care unit (ICU), total length of hospital stay and in-hospital mortality.

The number of patients was correlated to bacterial sepsis, surgical site infection and other infections, as well as in-hospital mortality between 2010 and 2017.

Results were presented as an absolute number and a percentage for the qualitative variables, as a mean ± standard deviation or median and interquartile range for the quantitative variables, in the most appropriate way.

The study was approved under the protocol number 2.963.344 for the FUNFARME / FAMERP ethics committee (nº CAAE: 00177818.8.0000.5415). There was no need for a free and informed consent term, since it involved only a collection of data from patients' electronic records, not having direct contact with participants.

## RESULTS

**Fig. 1:**
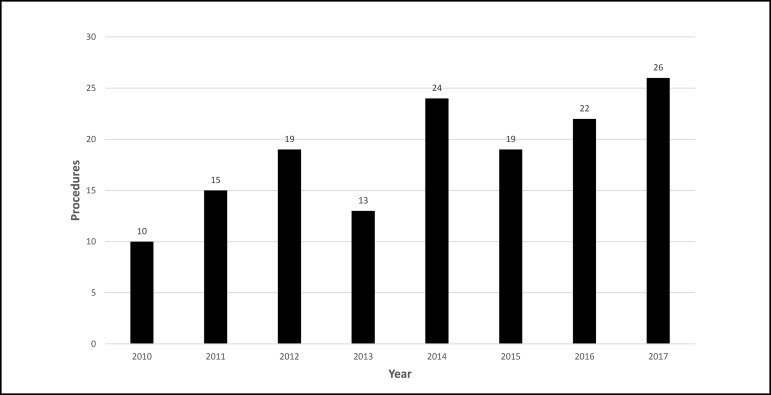
Number of procedures in patients with Down syndrome (DS) between the years 2010 to 2017.

The number of procedures per year was homogeneous (Figure 1), with 2017 being the year with the highest number of procedures (n=26) and 2010 with the lowest number of procedures (n=10), since there were only 6 months in the database for the year 2010. There was a slight prevalence of males over the females, 76 (51.7%) *vs*. 71 (48.3%). Three (2%) patients were younger than 30 days old, 97 (66%) were between 30 days and younger than 1 year old, and 47 (32%) were between 1 year and 17 years old, and 14 (9.5%) with a history of prematurity. The median weight was 5.8 kg (IQR 4.6 - 8.3).

For preoperative interventions, of 16 (10.9%) patients, 10 (6.8%) underwent mechanical ventilation and 6 (4.1%) required inotropic drugs before surgery.

Data on main diagnosis of CHD are presented in Table 1.

**Table 1:** Main pre-operative congenital heart defects (CHD) diagnosis.

Diagnosis	N (%)
Complete atrioventricular septal defect	55 (39.57%)
Ventricular septal defect	35 (25.18%)
Tetralogy of Fallot	14 (10.07%)
Atrial septal defect	13 (9.35%)
Partial atrioventricular septal defect	8 (5.76%)
Patent ductus arteriosus	7 (5.04%)
Complete atrioventricular septal defect (unbalanced)	3 (2.16%)
Coarctation of the aorta	2 (1.44%)
Tricuspid atresia	1 (0.72%)
Pulmonary stenosis (valvar and supravalvar)	1 (0.72%)
Total	139 (100%)

Evidence of pulmonary arterial hypertension (PAH) was reported in 15 (10.2%) of cases. Eight (5.4%) patients had non-cardiac abnormalities associated, such as Hirschsprung's syndrome, hypospadias, hypothyroidism and cleft palate.

The most prevalent procedures were complete AVSD repair with double patch technique 39 (26.53%), complete AVSD repair with "Australian technique" 13 (8.84%) and complete AVSD repair with single patch technique 6 (4.08%), totalizing 58 (39.45%), followed by VSD repair with bovine pericardial patch 29 (19.73%) and VSD repair with bovine patch with tricuspid valve plasty 7 (4.76%), totalizing 36 (24.49%), as shown in Table 2.

**Table 2:** Main surgical procedures performed in patients with Down syndrome (DS).

Surgical procedures	Percentage (%)	RACHS-1 categories
Complete AVSD repair with double patch technique	39 (26.53%)	3
VSD repair with bovine pericardial patch	29 (19.73%)	2
Partial AVSD repair with left AV valve cleft repair + ASD OP repair with bovine patch	11 (7.48%)	2
ASD repair with bovine patch	13 (8.84%)	1
Complete AVSD repair with "Australian technique"	7 (4.76%)	3
VSD repair with bovine patch + tricuspid valve plasty	7 (4.76%)	3
Double ligation of ductus arteriosus	7 (4.76%)	1
Complete AVSD repair with single patch technique	6 (4.08%)	3
Rastelli repair	5 (3.40%)	3
Modified Blalock-Taussig shunt repair	4 (2.72%)	3
Tetralogy of Fallot repair with transannular patch	4 (2.72%)	2
Tetralogy of Fallot repair with pulmonary valve ring preservation	3 (2.04%)	2
Pulmonary artery banding	3 (2.04%)	3
Glenn procedure + ASD enlargement	2 (1.36%)	4
Aortic arch reconstruction	2 (1.36%)	1
ASD enlargement + pulmonary artery banding + plasty of AV valves	1 (0.68%)	4
ASD enlargement + pulmonary artery banding + aortic arch reconstruction	1 (0.68%)	4
Tetralogy of Fallot repair + tricuspid valve plasty	1 (0.68%)	3
ASD repair + pulmonar valve plasty	1 (0.68%)	2
VSD repair + Aortic arch reconstruction	1 (0.68%)	2
Total	147 (100%)	

ASD=atrial septal defect; AV=atrioventricular; AVSD=atrioventricular septal defect; OP=*ostium primum*

CPB was performed in 129 (87.75%) surgical procedures, using a mean temperature of 28ºC, mean perfusion time of 95 minutes and mean aortic clamping time of 64 minutes.

According to the RACHS-1 adjusted surgical risk category, 22 (15%) procedures were placed into category 1, 49 (33.3%) in category 2, 72 (49%) in category 3 and 4 (2.7%) in category 4. There were no patients classified in risk categories 5 or 6 as shown in Table 3.

**Table 3:** Procedures according to RACHS-1 categories (Risk Adjustment for Congenital Heart Surgery).

RACHS-1 categories	Procedures (n)	Percentage (%)
1	22	15%
2	49	33.3%
3	72	49%
4	4	2.7%
Total	147	100%

There were no procedures classified as categories 5 or 6.

**Fig. 2:**
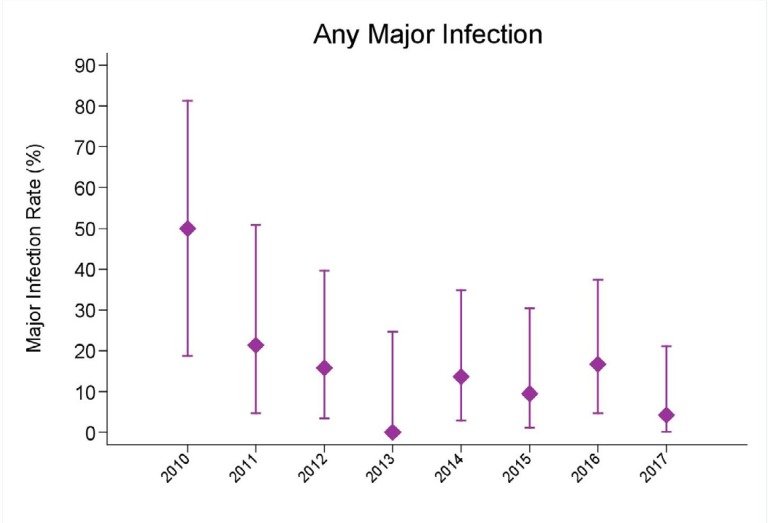
Comparison of postoperative results related to other infections between the years 2010 to 2017.

**Fig. 3:**
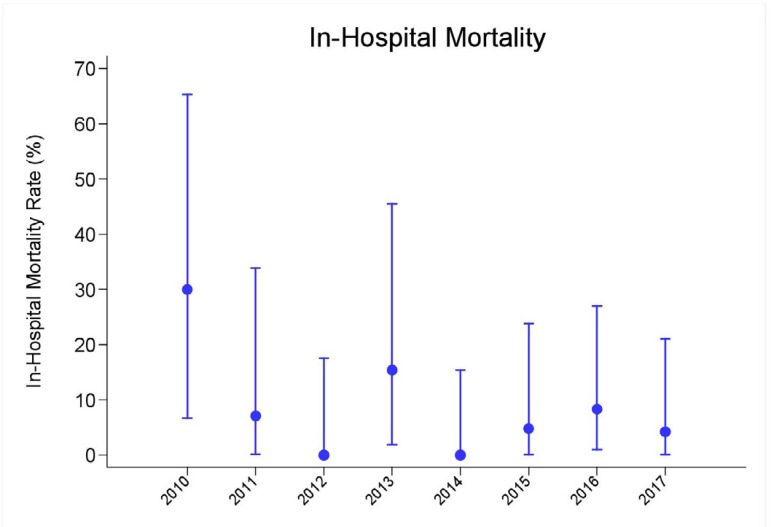
Comparison of postoperative results related to in-hospital mortality between the years 2010 to 2017.

Bacterial sepsis occurred in 10.2% of cases, surgical site infection in 6.1%, other infections in 14.3% and in-hospital mortality in 6.8%, between the years 2010 to 2017, as shown in Table 4 and Figures 2 and 3.

**Table 4:** Postoperative complications related to the number of patients between 2010 and 2017.

Complications	Year	Patients (n)	Results (n)	Percentage (%)
Bacterial sepsis	2010	10	4	40.0%
	2011	14	3	21.4%
	2012	19	2	10.5%
	2013	13	0	0.0%
	2014	22	3	13.6%
	2015	21	1	4.8%
	2016	24	1	4.2%
	2017	24	1	4.2%
	Total	147	15	10.2%
Surgical site infection	2010	10	4	40.0%
	2011	14	0	0.0%
	2012	19	1	5.3%
	2013	13	0	0.0%
	2014	22	0	0.0%
	2015	21	1	4.8%
	2016	24	3	12.5%
	2017	24	0	0.0%
	Total	147	9	6.1%
Other infections	2010	10	5	50.0%
	2011	14	3	21.4%
	2012	19	3	15.8%
	2013	13	0	0.0%
	2014	22	3	13.6%
	2015	21	2	9.5%
	2016	24	4	16.7%
	2017	24	1	4.2%
	Total	147	21	14.3%
In-hospital mortality	2010	10	3	30.0%
	2011	14	1	7.1%
	2012	19	0	0.0%
	2013	13	2	15.4%
	2014	22	0	0.0%
	2015	21	1	4.8%
	2016	24	2	8.3%
	2017	24	1	4.2%
	Total	147	10	6.8%

There was also a significant reduction in all complications and in-hospital mortality between the years, even with the increase in the number of patients operated, decreasing from 50% to 6.1%.

Time of ICU hospitalization and total postoperative hospitalization time are described in Table 5.

**Table 5:** Postoperative period intensive care unit and total length of stay.

	Median	IQR (25% - 75%)
ICU length of stay (days)	5	3 - 10
Total (days)	11	6 - 22

ICU=intensive care unit; IQR=interquartile range

## DISCUSSION

Since January 2002, 303 patients with CHD and DS were operated on. Understanding the need to obtain highly reliable data, it was opted to analyze data exclusively contained in the IQIC database which included 139 patients with CHD and DS after July 1, 2010.

About 50% of individuals with DS are affected by CHD^[1-3,7]^. The increase in prevalence of DS leads to an increase in number of patients with cardiac defects. A study published in the Annals of Thoracic Surgery in 2016 examined the Society of Thoracic Surgeons (STS) database and revealed the prevalence of DS in neonates undergoing cardiovascular surgery was 2.1%^[8]^.

Another meta-analysis published in 2017 on BMC Medical Genetics reported a higher prevalence of female patients with CHD and DS^[9]^. A different study published in 2016 in the Scientific Journal of the Medical Association - Acta Medica Portuguesa showed that 64.7% of patients were female^[7]^. A third study published in 2018 in the European Journal of Pediatrics also showed a higher prevalence of females (52.7%)^[10]^. The present study, however, revealed the opposite, with 51.7% of cases occurring in male patients.

More than half of procedures (68%) were performed in patients younger than one year of age. This data is in agreement with another study previously published in the Scientific Journal of the Medical Association - Acta Medica Portuguesa. This same study showed a similar data regarding the weight at the time of the operation as the median 6.4 kg (IQR 5.1 - 10.1), and in the present study the median was 5.8 kg (IQR 4.6 - 8.3)^[7]^.

The present study showed that the main CHD in DS patients was AVSD, in both complete and partial forms, present in 63 (45.32%) of diagnosis, followed by VSD with 35 (25.18%) cases. A Swedish study published in 2016 reported similar prevalence, with 42% AVSD and 22% VSD^[3]^. An Irish study published in 2018 also evidenced AVSD as more frequent, present in 30% of cases^[10]^. A third study in Germany evidenced the AVSD as more frequent followed by VSD, 51.2% and 25.1% respectively^[11]^.

A study published in 2007 in the Annals of Thoracic Surgery that included 10,032 patients who underwent CHD correction categorized according to RACHS-1 showed categories 2 and 3 as most common^[12]^. Data from the present study is in agreement, since 82.3% of the procedures also fit into categories 2 and 3. It is worth mentioning that according to Cavalcante et al.^[(13]^ although RACHS-1 has great ability to discriminate against mortality, this analysis, when performed in developing countries, also needs to take into account the associated clinical factors and structural and technological barriers.

Thus, it can be stated, according to the results of the present study and other previous studies, that the majority of the procedures performed in patients with DS are of RACHS-1 risk categories between 1 and 4, leaving the categories of high risk (5 and 6) as a minority or absent in many centers.

Left-right shunt or increased pulmonary blood flow CHD will certainly evolve with some degree of PAH^[14]^. The unadjusted AVSD and VSD are the main causes^[15]^. The present study demonstrated that 15 (10.20%) procedures were performed in patients already with some evidence of PAH, according to observations collected from surgical descriptions. When untreated, these patients can progress to Eisenmenger syndrome (ES), the final stage of PAH, when the right-left shunt is reversed, with high morbidity and mortality. In order to avoid this evolution, it has been recommended to operate these patients before 6 months of age, or even between 3 and 4 months in some centers^[14]^.

The presence of non-cardiac anomalies is six times higher in patients with DS compared to those without chromosomal anomalies, with gastrointestinal tract malformations being the most common. However, a Norwegian study published in 2018 in the Act Paediatrics revealed that in patients with DS and CHD, the association with extracardiac malformations was not as frequent^[16]^.

Even so, all cases of esophageal atresia and 10 of the 11 cases of Hirschsprung's disease reported occurred in patients with CHD. In the present study, no association was observed with esophageal atresia; however, one patient presented Hirschsprung's disease and another patient had already undergone correction of ileus atresia.

Infection is one of the complications most feared by surgeons and team in the postoperative period of cardiovascular surgery. This is due to the intimate relationship between presence of infection and increased mortality and length of hospital stay.

Sepsis is a much-feared postoperative complication due to the related mortality rate. Ma et al.^[17]^ published in 2007 that sepsis accounted for 11% of deaths in the postoperative period of congenital heart surgery. Barker et al.^[18]^ showed a sepsis rate of 2.6% in series with 30,078 cases. Our study evidenced that 15 (10.2%) procedures evolved with sepsis, however, the relation of these sepsis cases with in-hospital mortality was not performed. It is important to mention that the infection rate decreased from 40% in 2010 to 4.2% in 2017, as observed in Table 4.

Regarding surgical site infection (SSI), a study conducted at Boston Children's Hospital and published in the Annals of Thoracic Surgery in 2010 concluded that age younger than 1 year and CPB time greater than 105 minutes are independent risk factors for any type of SSI, as well as aortic clamping time greater than 85 minutes and at least 3 postoperative red blood cell transfusions as risk factors for the development of mediastinitis^[19]^. The Boston study's SSI rate was 2.16% (73 cases in 3367 procedures), lower than the total rate of the present study of 6.12% (9 cases out of 147 procedures), but no SSI was diagnosed in the year of 2017.

Barker et al.^[18]^ analyzed the STS database and found an infection rate of 2.8% (857 infections in 30,078 cases). They also reported a mortality rate of 22.2% in cases with infection against 3% in those without infection, and a longer hospitalization time of 21 days in 69.9% of the cases associated with the infection compared to 10.7% in cases without infection.

Patients with DS are more susceptible both to viral and bacterial infections and to the development of leukemia, and this has been attributed to the disordered immune system, which is one of the pathological features of the syndrome^[20]^. Non-immunological factors such as airway and ear abnormalities and also gastroesophageal reflux are also related to this susceptibility^[21]^.

Major immunological changes in this group of patients include reduction in T and B lymphocyte counts, absence of normal lymphocyte expansion in childhood, reduced size of the thymus, suboptimal response to immunizations, total and specific reduction of immunoglobulin A in saliva and neutrophils with decreased chemotaxis^[21]^.

This explains why this group of patients, even when submitted to procedures with lower levels of complexity, is affected with more infections than the general population.

The present study showed ICU hospitalization in the postoperative period with a median time of 5 days (IQR: 3-10), probably due to the reduced availability of ward beds. As a result of this problem, several patients who receive treatment in this center and are apt for discharge to the ward are sometimes discharged directly from the ICU to home.

The median time of total postoperative hospitalization observed in the present study was 11 days (IQR: 6 - 22) and this can be related to the infection rate in the postoperative period, which, as is scientifically proven, significantly increases postoperative in-hospital length of stay, even though the study did not correlate infection cases with increased length of hospitalization specifically.

Regarding mortality of DS patients submitted to CHD repair, Dias et al.^[7]^ reported a rate of 2.9% (3/102 cases) in 30 days. The mortality rate of the STS database for pediatric patients undergoing cardiovascular surgery was 4%, not specifying patients with and without DS. In the present study, there were 10 (6.8%) intra-hospital deaths among the procedures analyzed, being 4.2% in 2017, but still higher than those reported in previous studies^[12]^.

### Limitation

The limitations of the study are related to single center data analysis and non-correlation of infection cases that resulted in increased hospitalization and in-hospital mortality.

## CONCLUSION

Surgical treatment in patients with CHD and DS usually does not require highly complex surgical procedures, but are affected by infectious complications, resulting in a longer ICU and hospital length of stay with considerable mortality.

**Table:** 

Authors' roles & responsibilities	
FCGBS	Substantial contributions to conception or design of the work; or the acquisition, analysis, or interpretation of data for the work; final approval of the version to be published
UAC	Substantial contributions to conception or design of the work; or the acquisition, analysis, or interpretation of data for the work; final approval of the version to be published
CHM	Final approval of the version to be published
ANM	Final approval of the version to be published
JDPB	Final approval of the version to be published
BCB	Drafting of the work or revising it critically for important intellectual content; final approval of the version to be published
RGF	Final approval of the version to be published
MFG	Drafting of the work or revising it critically for important intellectual content; final approval of the version to be published
